# Finding semantic patterns in omics data using concept rule learning with an ontology-based refinement operator

**DOI:** 10.1186/s13040-020-00219-6

**Published:** 2020-09-01

**Authors:** František Malinka, Filip železný, Jiří Kléma

**Affiliations:** 1 Department of Computer Science, Czech Technical University in Prague, Karlovo náměstí 13, Prague, 121 35 Czech Republic; 2 Czech Centre for Phenogenomics, Institute of Molecular Genetics of the Czech Academy of Sciences, Prague, Czech Republic

**Keywords:** Symbolic machine learning, Enrichment analysis, Ontology, Taxonomy, Gene expression, Biclustering

## Abstract

**Background:**

Identification of non-trivial and meaningful patterns in omics data is one of the most important biological tasks. The patterns help to better understand biological systems and interpret experimental outcomes. A well-established method serving to explain such biological data is Gene Set Enrichment Analysis. However, this type of analysis is restricted to a specific type of evaluation. Abstracting from details, the analyst provides a sorted list of genes and ontological annotations of the individual genes; the method outputs a subset of ontological terms enriched in the gene list. Here, in contrary to enrichment analysis, we introduce a new tool/framework that allows for the induction of more complex patterns of 2-dimensional binary omics data. This extension allows to discover and describe semantically coherent biclusters.

**Results:**

We present a new rapid method called sem1R that reveals interpretable hidden rules in omics data. These rules capture semantic differences between two classes: a target class as a collection of positive examples and a non-target class containing negative examples. The method is inspired by the CN2 rule learner and introduces a new refinement operator that exploits prior knowledge in the form of ontologies. In our work this knowledge serves to create accurate and interpretable rules. The novel refinement operator uses two reduction procedures: Redundant Generalization and Redundant Non-potential, both of which help to dramatically prune the rule space and consequently, speed-up the entire process of rule induction in comparison with the traditional refinement operator as is presented in CN2.

**Conclusions:**

Efficiency and effectivity of the novel refinement operator were tested on three real different gene expression datasets. Concretely, the Dresden Ovary Dataset, DISC, and m2816 were employed. The experiments show that the ontology-based refinement operator speeds-up the pattern induction drastically. The algorithm is written in C++ and is published as an R package available at http://github.com/fmalinka/sem1r.

## Background

Nowadays, omics data analysis that integrates semantics in the form of external prior knowledge with raw measurements is becoming more and more popular in computational biology [1–3]. A typical example of integrative gene expression data analysis may deliver a direct link between a phenotype and existing annotation terms at different levels of generality. The integration helps scientists to interpret gene expression data easier because it can reveal gene sets that share common biological properties. Semantic data are stored in databases, oftentimes in an ontology format. In this area, an important role is played by The Open Biological and Biomedical Ontology (OBO) Foundry [4], which provides validation and assessment of ontologies to ensure their interoperability. Dozens of ontologies from various biological domains can be downloaded from http://www.obofoundry.org/.

### Gene set enrichment analysis

One of the most popular methods that uses this type of semantics utilizing a connection between ontologies or gene set databases and genes is *enrichment analysis*, *Gene Set Enrichment Analysis* (GSEA) [5] represents one of its most frequently used implementations. The enrichment analysis identifies a list of significantly enriched ontological terms from a provided list of differentially expressed genes that is sorted according by some ranking metric (p-value, log fold change, etc.). To discover a certain molecular function or biological process that is shared over the set of differentially expressed genes, Gene Ontology [6, 7] is an appropriate and often used annotation database. GSEA overcomes certain limitations of the statistical enrichment assessment based on hypergeometric, *χ*^2^, or Fisher exact test, namely the information loss caused by selection of significant genes before the enrichment analysis. An example of GSEA outcome that is induced from data over the KEGG database can be the following:


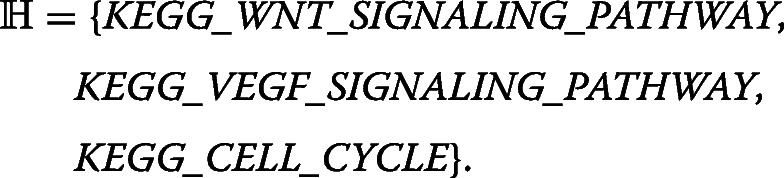


In our view, this GSEA outcome corresponds to a hypothesis that can be seen as a collection of three simple rules where each rule has length one and says, independent of the other rules, that the corresponding term in the rule is significantly enriched in the reported set of genes against a background/control gene set. Unfortunately, GSEA in particular, and enrichment analysis in general, cannot produce more complex hypotheses. For example, the hypothesis above does not say that *KEGG_WNT_SIGNALING_PATHWAY* and *KEGG_CELL_CYCLE* are enriched simultaneously, in conjunction. The form of hypothesis only says that these terms are enriched individually. On the other hand, let *R* be the following rule:


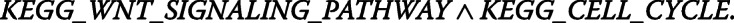


*R* says that simultaneous occurrence of the terms *KEGG_WNT_SIGNALING_PATHWAY* and *KEGG_CELL_CYCLE* in the annotation of a gene (frequently) leads to its upregulation. The upregulation score for the rule *R* is computed from a gene set where each gene has to be associated with both terms simultaneously. In our framework, and unlike the traditional enrichment analysis, we will be able to cope with these conjunctive rules.

Moreover, the dimension of biological samples/conditions is disregarded in the enrichment analysis, only the dimension of genes is taken into consideration when constructing annotations. The enrichment analysis supposes a gene set of interest (e.g. genes that are differentially expressed) to be a part of the input. Consequently, these methods can only be applied in such biological experiments, where samples are split into two groups, treatments and controls. However, the treatment and control labels are often not available. In most cases, the split into groups is unclear, the sample groups may overlap or form complex taxonomies. Under these conditions, any set of differentially expressed genes cannot easily be determined. For this reason, we suppose that samples are described with a rich ontology of annotation terms (locations, conditions, complex treatments, etc.) and bring an opportunity to further generalize the rules with extra terms from this ontology that can be added into the rules. This allows for inducing a rule that self-defines the semantically coherent joint groups of genes and samples; the genes tend to be upregulated in the sample group. The induction is fully automated and driven by the context provided in the measurements and annotation ontologies. In other words, GSEA uses a 1-dimensional space of genes to induce a list of significantly enriched annotation terms. In this work, we expand onto 2-dimensional expression space and consequently allow for generation of hypotheses that represent a set of genes upregulated in a specific set of samples/biological conditions. An example of the hypothesis could be the following rule:


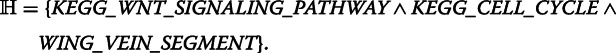


This hypothetical example shows the case where genes belonging to *KEGG_WNT_SIGNALING* and *KEGG_CELL_CYCLE* pathways are frequently upregulated in samples from *WING_VEIN_SEGMENT*, which makes a specific body part of Drosophila melanogaster.

### Rule learning with ontological background knowledge

We use rule learning [8, 9] to construct the above-outlined hypotheses. Rule learning refers to a class of supervised machine learning methods that induce a set of classification rules from a given set of training examples. For a binary task, training examples are assigned to two disjoint sets of positive and negative examples. The rule is an if-then statement where the antecedent is in the form of a conjunction of positive or negative logical terms, and the consequent is a class label. The final decision regarding an unseen example is provided by a set of rules or their ordered list. The rules are widely used in the fields of medicine and biology for their easy and clear interpretation [10–12] contrary to neural networks, for instance.

As previously mentioned, one of the things that can help scientists interpret their data in a more natural way is background knowledge. Bioinformatics frequently deals with Gene Ontology [6, 7] and there are other types of structured databases, such as KEGG [13–15], which can also be interpreted as an ontology or a taxonomy. Medicine employs Disease Ontology [16, 17] or SNOMED-CT, natural language processing makes use of WordNet [18] or YAGO [19], dedicated ontologies are often encountered in industry too.

In our work, these two concepts, rule learning and ontologies or taxonomies, are combined. We observed that the ontologies reasonably increase accuracy and robustness of induced rules. However, they also reasonably raise the number of logical terms available for rule construction, which consequently leads to prohibitive growth of hypothesis space and inefficiency of rule learning. This inefficiency can reasonably be reduced with consistent utilization of the known hierarchical relationships between the ontology terms that cannot be handled with the traditional rule learning methods [20, 21]. In this paper, we will focus on the binary task (positive and negative examples, two classes only) and multiple rule models (the output of the learning algorithm is multiple rules).

The main motivation for this paper was our work published in [22], in which we introduced a technique called *semantic biclustering*. This type of biclustering infers a human easily readable form of hypothesis describing only a single target class (also known as the target concept). This technique is applied to a gene expression data where highly expressed genes in corresponding samples are considered as the target class. One of the proposed methods solves the problem of semantic biclustering by linearizing a two-dimensional binary data matrix and a set of ontologies to an attribute-value representation that can be figured out using one of the well-known rule learning algorithms such as CN2 [20, 23], RIPPER [21], or PRIM [24]. However, current ontologies, such as Gene Ontology, contain tens of thousands of hierarchically ordered terms. As a result, building a classification model without a preprocessing step is time and memory consuming. For this reason, we introduce a new refinement operator for a rule learning algorithm that examines properties between given data, ontologies, and its mutual relations to speed-up and improve the process of learning.

One of the related subfields of machine learning that can exploit formalized prior knowledge such as ontologies or taxonomies is Inductive Logic Programming (ILP) [25] where a key challenge is to prune the search space of (first-order logic) rules. For its ability to work with this form of prior knowledge, we were inspired by this subfield. In [26], the authors proposed a refinement operator to construct conjunctive relational features. This algorithm uses taxonomies to exclude conjunction from the exploration process if the conjunction contains a feature together with any of its subsumees. In [27], the authors find and prune such hypotheses that are equivalent to a previously considered hypothesis. To test such equivalency in given domain theory, they proposed a saturation method for a first-order logic clause with the property that two clauses are equivalent whenever their saturations are isomorphic.

However, the highly expressive first-order logic setting of ILP is traded off by high computational demands and high complexity of resulting patterns. The latter presents a challenge when interpreting and validating the outputs. For the analysis task addressed here, the expressiveness and complexity of ILP is unnecessary. We thus seek to design an efficient rule-refinement operator in the simpler setting of IF-THEN rules corresponding to propositional-logic formulas.

### Propositional rule learning

We base our approach on the classical rule learning algorithm CN2 [20]. The input to CN2 is an attribute-value description of a set of examples along with the class labels of the examples, i.e. the *training set*. The output is a set of rules predicting class labels from the attribute values. Each rule has the form

1 $$ a_{1} = v_{1} \wedge a_{2}=v_{2} \wedge \ldots \rightarrow {class}  $$

where *a*_*i*_ denote attributes as defined in the example set, *v*_*i*_ are values assumed for the prediciton, and *class* is the predicted class. For each *class*, the algorithm first considers an empty set of conditions on the left-hand side. Such a rule will trivially predict *class* for all examples, which will typically be incorrect. The set of conditions thus needs to be iteratively extended until the rule has sufficient quality, i.e., it avoids enough out-of-class examples while retaining the class prediction for sufficiently many in-class examples. The addition of a condition into a rule is called rule refinement. An applied refinement may turn out unsuitable even with additional refinements, a high-quality rule is not found. Thus the algorithm can backtrack and search an alternative refinement. The exact succession of these operations is prescribed by the Beam search heuristic [28]. When a rule is accepted and the training set still contains *class* examples not predicted by it, a new rule is searched. The loop terminates when each positive example is predicted positive by at least one of the accepted rules.

## Methods

We aim to learn rules similar in form to (1), except each condition on the left-hand side will correspond to an assumed ontological term. Thus the logical conjunction will simply correspond to a set of terms. Rules will be searched only for the positive class, as any example not classified as positive is deemed negative by default (we work in the binary classification setting). Thus the *class* symbol in all rules will indicate the positive class, and we can drop the right-hand side of rules. Therefore, a rule in our context is simply a set of terms.

Our goal is to find a set of rules which fit well a supplied training set as described above in the context of the CN2 algorithm. To this end, we introduce a special refinement operator that, due to the taxonomic nature of the assumed conditions, significantly reduces the search space of rules and consequently reduces run times of the rule learner in comparison to the traditional refinement operator without a loss of accuracy. For example, if term *t*1 is in the rule and the ontology prescribes that *t*2 is more general than *t*1 then adding *t*2 to the rule is obviously useless. We can thus safely prune from the search space all rules combining *t*1 and *t*2.

Technically, the proposed ontology-based refinement operator uses two reduction procedures: a *Redundant Generalization* that omits candidate rules based on a relation generalization-specialization and a *Redundant Non-potential* that omits the candidate rules which cannot improve classification accuracy.

### Problem formalization

To describe our rule-learning algorithm in detail, we first define a few formal concepts. We are given

- Two sets *E*^+^,*E*^−^ of positive and negative (respectively) examples.
- A set *T* of ontological terms with a partial order ≽ which encodes the “more general than" relation. For example, with *t*1=*biological process* and *t*2=*developmental process*, we have *t*1≽*t*2.
- An annotation function *M* which maps each example to a subset of *T*, i.e. *M*:*E*^+^∪*E*^−^→2^*T*^.

From *M*, we can derive a reverse mapping *M*^′^:*T*→2^*E*^ producing the set of examples annotated with a given term, i.e. *M*^′^(*t*)={*e*∈*E*:*t*⊆*M*(*e*)}. It is also useful to define the transitive closure *S*(*t*) of *M*^′^(*t*) as the set of all examples annotated by *t* or any term less general than *t*, i.e.

2 $$ S(t) = \bigcup_{t' \in T, t \succeq t'} M(t')  $$

If *t* is the only term in a rule, then *S*(*t*) is the set of all examples for which the rule predicts the positive class. *S*(*t*) is also called the *cover* of the rule. More generally, for a rule conjoining an arbitrary set *R*⊆*T* of terms, we define the *cover function* as

3 $$ \Theta(R) = \bigcap_{t \in R} S(t)  $$

Finally, we define a generality relation ≽_*r*_ on rules. Let *R*1,*R*2⊆*T*, then *R*1≽_*r*_*R*2 if and only if *Θ*(*R*1)⊇*Θ*(*R*2).

#### **Example 1**

**Fig. 1:**
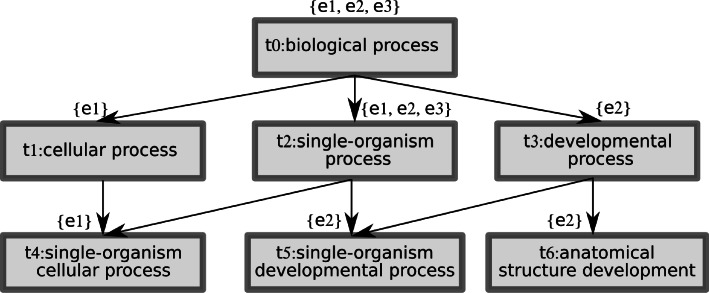
An example of partial-order binary relation ≽ over a set of terms *T*. The partial-ordered set is depicted in the form of a Hasse diagram. The terms and relations come from Gene Ontology. Elements in curly brackets represent examples that are associated with the individual terms according to the mapping *S*. In other words, the information about associations between examples and terms captured in the mapping *M*^′^(*t*) has already been hierarchically spread over the whole ontology

Consider 3 hypothetical examples and 7 actual ontology terms as shown in Fig. 1. The term generality relation ≽ corresponds to the direction of edges from more to less general. Here we have *M*(*e*_1_)={*t*4},*M*(*e*_2_)={*t*5,*t*6},*M*(*e*_3_)={*t*2}. *M*^′^(*t*) is shown above each *t* box. Finally, *S*(*t*)=*M*^′^(*t*) for *t*∈{*t*4,*t*5,*t*6} but e.g. *S*(*t*1)=*M*^′^(*t*1)∪*M*^′^(*t*4)={*e*1}.

### Proposed algorithm

The algorithm proposed in this work induces a hypothesis from data in the form of a set of rules. To induce a hypothesis consisting of more rules we apply a covering algorithm that has its origin in the AQ family of algorithms [29] and it is also used in CN2. The covering algorithm consists of two steps: (1) induce a single rule from the current set of examples, (2) exclude the examples that are covered by this single rule from the current set of examples; these two steps are iteratively applied starting with the set of all examples until all positive examples are covered or a certain number of induced rules is reached. This process is described in Algorithm 1 and that algorithm we refer to as *sem1R*. As an input, the following data are required: a set of positive *E*^+^ and negative *E*^−^ examples, a set of ontologies $\mathcal {O}$, and a maximal size of the set of induced rules *k*. An output is a set of induced rules. An *induceSingleRule* function returns the best rule based on selected evaluation function. The function *induceSingleRule* is described in Algorithm 2, all evaluation functions can be found in the “Evaluation criteria” section.

Contrary to CN2, the *sem1R* algorithm has the relations over terms that are explicitly specified in provided ontologies. Intuitively, if this kind of knowledge were exploited then we would expect some benefits during the process of inducing rules because the structure of terms is known. In this case, the main benefit is speeding up the process of inducing rules and removing obvious redundancy between the terms in rules. This was the main motivation for the following reduction procedures. 
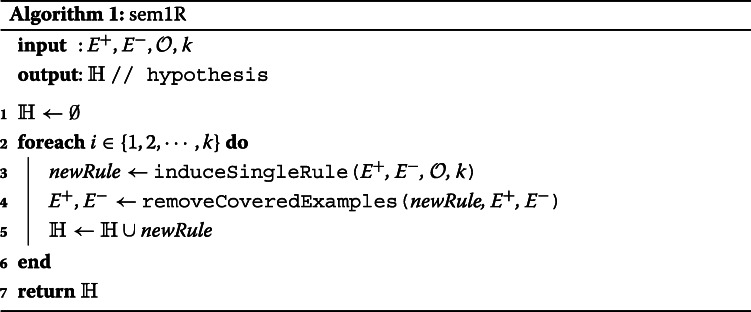


#### Reduction procedures

In this section, we formulate two procedures that significantly reduce a rule space in comparison with the traditional rule learning methods such as CN2.

#### Redundant generalization

This reduction method eliminates such terms occurring in a rule which are more general than any other term of the rule. Such terms in the rule do not affect a set of examples covered by the rule and consequently do not change its impact. Evidently, the set of covered examples is only affected by the most stringent sets of examples according to the mapping *S*.

##### **Theorem 1**

Let *R*1 be a rule and suppose that term *t*1∈*R*1 and a term *t*2∈*R*1 where *t*1 is more general than *t*2. Then, the rule *R*1 covers an equal set of examples as a rule $\overline {R1} = R1 \backslash \{t1\}$ that does not contain *t*1:

$$\Theta(\overline{R1}) = \Theta(R1)$$

and the rule *R*1 is called a *redundant generalization* of $\overline {R1}$.

##### *Proof*


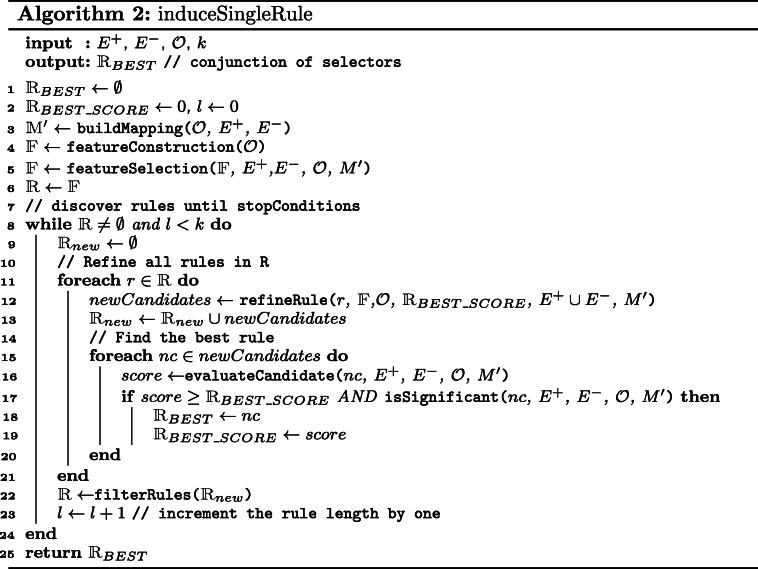


For simplicity, we take into consideration only rules with cardinality 1. Given this, mapping *S* can be seen as a cover operator *Θ* because it only makes an intersection over all sets of examples according to *S*. Also, a rule of cardinality 1 will be denoted as a term because we do not want to distinguish the relations over the set of terms and the set of rules. In this case, the ≽ relation over terms is equivalent to ≽_*r*_ relation over rules. This simplification does not lose generality.

A term cannot be associated with a higher number of examples than its more general counterpart. Concurrently, examples associated with a more specific term make a subset of examples associated with a more general term, written as *t*1≽*t*2⇒*S*(*t*2)⊆*S*(*t*1) where *t*1,*t*2∈*T*. Now, let rule *R*1={*t*1,*t*2} consist of two terms such that *t*1≽*t*2 and rule $\overline {R1} = \{t2\}$ consists of only term *t*2. Then *R*1 covers an equal set of examples as $\overline {R1}$. This equality is proven below.

$$\Theta(R1) = \Theta(\overline{R1})$$

$$S(t1) \cap S(t2) = S(t2)$$

$$\{e \in E: S(t2) \subseteq S(t1)\} = S(t2)$$

$$S(t2) = S(t2).$$

□

##### **Example 2**

Consider the ontology *O* and mappings *M*,*M*^′^,*S* from Example 1. Let rule *R*1={*t*0,*t*2}, term *t*0 is more general than *t*2 (*t*0≽*t*2) and this rule covers examples *e*1,*e*2,*e*3 because *Θ*(*R*1)=*Θ*({*t*0,*t*2})=*S*(*t*0)∩*S*(*t*2)={*e*1,*e*2,*e*3}. Now, consider a rule $\overline {R1} = \{t2\}$ that also covers examples *e*1,*e*2,*e*3 since $\Theta (\overline {R1}) = S(t2) = \{e1, e2, e3\}$ and as we can see, term *t*0 occurring in the rule *R*1 does not influence a set of covered examples. Given this, rule *R*1 covers the same set of examples as rule $\overline {R1}$. For this reason, rule *R*1 is Redundant Generalization and rule $\overline {R1}$ is not Redundant Generalization.

To achieve a non-Redundant Generalization rule, i.e. the rule where the relation ≽ does not exist between any terms in the rule, we have to apply Redundant Generalization procedure until the relation ≽ between terms in the rule has not been found. As we can see in Example 2, this reduction procedure decreases the cardinality (length) of the rules.

#### Redundant non-potential

In the previous case, the Redundant Generalization method reduces a rule space as a result of its ability to decrease the cardinality of rules. Specifically, this reduction capability is applied to the refinement operator that gradually extends rules by adding new terms into them. Redundant Generalization method can generate fewer candidate rules because terms that are in a relation with another term are not appended to the refined rule.

Contrary to the previous method, the Redundant Non-potential method does not utilize relations among terms to reduce a rule space but compares rules with each other and removes such rules that cannot reach a higher quality value than the current best rule has. The ability to recognize non-potential rules can be used for a direct reduction of rules in a rule space and also for eliminating a number of candidate rules in a process of rules refining. Firstly, we define two types of evaluation function: *Q* evaluating a quality of rule based on the number of covered/uncovered examples, and *Q*_*p*_ that evaluates a potentially maximum quality of rule that could possibly be achieved over its future refinements. Examples of *Q* functions are depicted in Eqs. 11, 13, and 15. Corresponding *Q*_*p*_ functions are depicted in Eqs. 12, 14, and 17. For the moment, we can say that *Q*_*p*_ function expresses an upper boundary of a rule quality. This upper bound can be reached when we know that rule refinements can only reduce the set of examples the rule covers. Then, the best potential refinement does not lose any positive examples from the current cover while ceasing to cover all the current negative examples. A Redundant Non-potential rule and all its more specific rules can be safely disregarded in the single rule induction process because there is a guarantee that these rules cannot exceed an upper boundary of the rule quality represented by *Q*_*p*_.

To illustrate, consider an arbitrary rule *R*1 and its more specific rule *R*2 (*R*1≽_*r*_*R*2) which was created by refinement operator application. *R*2 covers a subset of examples covered by *R*1 (*Θ*(*R*2)⊆*Θ*(*R*1)). Unfortunately, ACC or F1-score are not monotone functions, meaning that it is not guaranteed that *R*2 must always have a higher ACC or F1-score than *R*1. For this reason, *R*2 cannot be safely pruned from a rule space because it is not obvious whether other refinements of *R*2, which are more specific than *R*2, can potentially achieve a higher score than *R*1 even though *R*2 could have a worse score than *R*1. To prune the rule space safely, we maintain the upper bound of rule quality *Q*_*p*_. Given this, if rule *R*2 (refinement of *R*1) has a lower *Q*_*p*_ value than *R*1’s value of *Q* then *R*2 is a *Redundant Non-potential* and this rule, along with all its more specific extensions/refinements, can be safely pruned from a rule space.

##### **Theorem 2**

Let $\mathcal {R} = <R, \succeq _{r}>$ be a quasi-ordered set representing a rule space, where *R*={*R*1,*R*2,*R*_*best*_}. Binary relation ≽_*r*_ is defined on *R*1 and *R*2 as ≽_*r*_={(*R*1,*R*2)} meaning that *R*2 is more specific than *R*1; relation of *R*_*best*_ is disregarded - may be arbitrary. If potential quality (*Q*_*p*_) of the rule *R*1 is smaller than the quality *Q* of rule *R*_*best*_ then the rule *R*1 and all its potential more specific rules, i.e. *R*2, can be pruned from the set of rules *R* thus from the rule space $\mathcal {R}$. Then the rules *R*1 and *R*2 are called *Redundant Non-potentials*.

##### *Proof*

First of all, suppose that a target class is represented by positive examples. Secondly, suppose an evaluation function whose highest value is returned when all positive examples and none of the negative examples are covered. An example of this function can be ACC or F1-score. Note, that ACC is given by equation *TP*+*TN*/(*TP*+*TN*+*FP*+*FN*) (see the “Evaluation criteria” section) and the reason, why we affect only *TP* and not *TN*, is simple. An example that is classified as *TP* has to be covered by a rule. On the other hand, an example classified as *TN* does not have to be covered by a rule. Since we focus on the target class, an arbitrary rule reaches a higher score if a new rule covers the same set of positive examples as a rule and does not cover any other negative example. □

##### **Example 3**

Consider the ontology *O* and mappings *M*,*M*^′^,*S* from Example 1, and two rules *R*1={*t*2} and *R*2={*t*3}. Further, we define a set of positive examples *E*^+^={*e*1,*e*3} and a set of negative examples *E*^−^={*e*2}. Firstly, we evaluate the quality of the rules according to ACC measure (see Eq. 11)

4 $$\begin{array}{@{}rcl@{}}  {Q_{ACC}(R1) = \frac{TP + TN}{TP + TN + FP + FN} = \frac{2 + 0}{2 + 0 + 1 + 0} = \frac{2}{3}} \end{array} $$

5 $$\begin{array}{@{}rcl@{}} Q_{ACC}(R2) = \frac{TP + TN}{TP + TN + FP + FN} = \frac{0 + 0}{0 + 0 + 1 + 2} = 0 \end{array} $$

Now, we compute a potential quality score of *R*2 (see Eq. 12):

6 $$\begin{array}{@{}rcl@{}}  Q_{p\_ACC}(R2) = \frac{TP + TN + FP}{TP + TN + FP + FN}= \frac{0 + 0 + 1}{0 + 0 + 1 + 2} = \frac{1}{3} \end{array} $$

Evidently, the potential quality of *R*2 is smaller than the quality of *R*1 so we can exclude the rule *R*2 and all its more specific rules (e.g. {*t*5,*t*6}) from the rule space. Note that an example of how to compute evaluation measures can be found in the next section.

To achieve the most effective pruning of rule space, we store a value of the highest quality rule that has been discovered during the learning process in $\mathbbm {R}_{BEST\_SCORE}$ variable, see Algorithm 2. If the potential quality (*Q*_*p*_(*R*)) of currently examined rule *R* is less than the $\mathbbm {R}_{BEST\_SCORE}$, then the rule *R* and all its more specifics rules are *Redundant Non-potential* and can be excluded from a rule space.

### Evaluation criteria

It is necessary to know the quality of each rule because the rule with the highest value is needed for the final hypothesis. In this case, we define three evaluation functions: accuracy (ACC), F1-score (F1), area under the ROC curve (AUC), and their adjusted versions for evaluating the potentially best results that the current rule can achieve after refinements in future evaluations. Accuracy works well for balanced problems (the number of positive examples is similar to the number of negative ones) and both classes are equally important. F1 and AUC help when dealing with imbalanced classes, F1 puts more emphasis on the positive class.

**Fig. 2:**
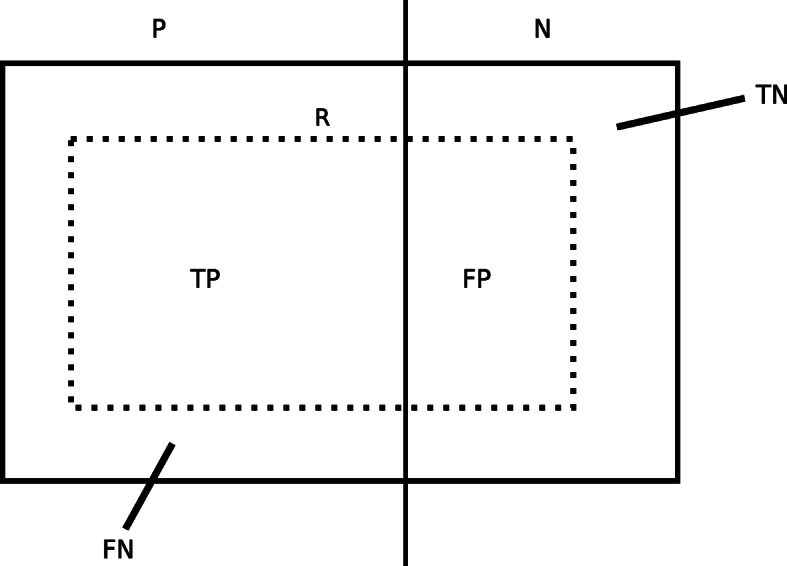
A graph representing a set of positive examples *P* and negative examples *N* and the way they are covered by a rule *R* assuming that *R* is focused on the classification of positive examples. Subspaces corresponding to TP, FP, FN and TN examples are also depicted

First of all, we define four elements of confusion matrix: number of true positives (TP), number of false positives (FP), number of false negatives (FN), and number of true negatives (TN) examples that are covered by an arbitrary rule *R*, see Fig. 2.

TP is given as a cardinality of the intersection of two sets, a set of examples that are covered by the rule *R* and a set of positive examples *E*^+^. FP is given as a cardinality of the intersection of two sets, a set of examples that are covered by the rule *R* and a set of negative examples *E*^−^. TN is given as a cardinality of the subtraction of two set, a set of negative examples *E*^−^ and a set of examples that are covered by the rule *R*. Finally, FN is given as a cardinality of subtraction of two sets, a set of positive examples *E*^+^ and a set of examples that are covered by the rule *R*. All equations are shown below.

7 $$\begin{array}{@{}rcl@{}}  TP = |\Theta(R) \cap E^{+}| \end{array} $$

8 $$\begin{array}{@{}rcl@{}} FP = |\Theta(R) \cap E^{-}| \end{array} $$

9 $$\begin{array}{@{}rcl@{}} TN = |E^{-} \backslash \Theta(R)| \end{array} $$

10 $$\begin{array}{@{}rcl@{}} FN = |E^{+} \backslash \Theta(R)| \end{array} $$

Corresponding accuracy (ACC) of an arbitrary rule *R* can be computed by the widely known equation below:

11 $$\begin{array}{@{}rcl@{}}  Q_{ACC}(R) = \frac{TP + TN}{TP + TN + FP + FN} \end{array} $$

However, the potentially highest accuracy of rule refined from *R* is computed differently. In Eq. 11, we see that the eventual accuracy is given by the numerator (TP and TN) whereas the denominator has the normalization function. The refinement may improve the rule quality in such a way that the examples that are classified as FP will be re-classified to TN, i.e. the numerator of $Q_{p\_ACC}$ may at best be given by the sum of TN, TP, and FP. The equation for the potentially highest quality reached through refinement follows:

12 $$\begin{array}{@{}rcl@{}}  Q_{p\_ACC}(R) = \frac{TP + TN + FP}{TP + TN + FP + FN} \end{array} $$

The computation of $Q_{p\_ACC}$ in Eq. 12 assumes that the rule *R* aims to cover positive examples rather than negative ones. In other words, examples that are covered by the rule *R* are classified as positive. Secondly, we propose another evaluation measure that is based on *F*1-score that implicitly does not take into account the number of TNs. Its common form is depicted in Eq. 13.

13 $$\begin{array}{@{}rcl@{}}  Q_{F1}(R) = \frac{2 \times TP}{2 \times TP + FP + FN} \end{array} $$

The corresponding version of potentially best accurate rule created by applying refinement operator to rule *R* that is based on the *F*1 measure takes the following form:

14 $$\begin{array}{@{}rcl@{}}  Q_{p\_F1}(R) = \frac{2 \times TP}{2 \times TP + FN} \end{array} $$

where all negative examples covered by rule *R* (FP) are excluded from the denominator in comparison with Eq. 13. Since there is still the possibility of finding such a rule which covers all examples determined as TP and none of the FPs.

#### **Example 4**

Consider the ontology *O* and mappings *M*,*M*^′^,*S* from Example 1, and a set of positive (*E*^+^) and negative (*E*^−^) examples from Example 3. Further, we define a rule *R*={*t*2}. First of all, we find examples that are covered by the rule using *Θ* operator, i.e. *Θ*({*t*2})=*S*(*t*2)={*e*1,*e*2,*e*3}. Secondly, we compute TP, FP, FN and TN:

$$TP = |\Theta(r) \cap E^{+}| = |\{e1, e2, e3\} \cap \{e1, e3\}| = 2$$

$$FP = |\Theta(r) \cap E^{-}| = |\{e1, e2, e3\} \cap \{e2\}| = 1$$

$$TN = |E^{-} \backslash \Theta(r)| = |\{e2\} \cap \{e1, e2, e3\}| = 0$$

$$FN = |E^{+} \backslash \Theta(r)| = |\{e1, e3\} \cap \{e1, e2, e3\}| = 0$$

Finally, we substitute these numbers in Eqs. 11 and 12:

$$Q_{ACC}(R) = \frac{TP + TN}{TP + TN + FP + FN} = \frac{2 + 0}{2 + 0 + 1 + 0} = \frac{2}{3}$$

$$Q_{p\_ACC}(R) = \frac{TP + TN + FP}{TP + TN + FP + FN}= \frac{0 + 0 + 1}{0 + 0 + 1 + 2} = \frac{1}{3}$$

The final ACC of rule *R* over the set of positive and negative examples is $\frac {2}{3}$ and the potential best ACC for the set rule and the set of examples is $\frac {1}{3}$.

Finally, let us give the rule quality in terms of AUC. The area under the curve can be computed easily. Since only the single rule is taken into consideration, its quality is determined by a single point in the ROC plot and it can be computed as a sum of areas of two triangles and one rectangle using an Eq. 15.

15 $$\begin{array}{@{}rcl@{}}  Q_{AUC}(R) = FPR \times TPR + (1-FPR) \times TPR + \frac{(1-FPR) \times (1-TPR)}{2} \end{array} $$

TPR (true positive rate) and FPR (false positive rate) are calculated as follows:

16 $$\begin{array}{@{}rcl@{}}  TPR=\frac{TP}{TP+FN}, FPR=\frac{FP}{FP+TN} \end{array} $$

17 $$\begin{array}{@{}rcl@{}}  Q_{p\_AUC}(R) = TPR + \frac{(1-TPR)}{2} \end{array} $$

The adjusted version of AUC computing a potentially best AUC that a rule can achieve is shown in Eq. 17. In contrast to Eq. 15, $Q_{p\_AUC}$ supposes that FPR goes to zero.

### Feature construction

In the Problem definition section, we defined the rule space $\mathcal {R}$ as a quasi-ordered set that is expressed as a pair of a set of rules and the relation ≽_*r*_ between rules. In addition, the form of rules is determined by propositional logic; more precisely, the rule is restricted to a conjunction of positive terms, i.e.

$$R=t1 \wedge t2 = \{t1,t2\}, t1,t2 \in O.$$

The first step in the rule learning process is feature construction because rule learning employs features as their basic building blocks. In this work, features are constructed trivially from a set of terms *T* which comes from the ontology *O* where each ontology term corresponds to one feature.

### Feature selection

Oftentimes, a constructed feature set is extremely large and also redundant since it contains many features that are not associated with any example. For this reason, a feature selection method is highly recommended. Given this, we propose three various feature selection methods.

#### FS_atLeastOne

The first feature selection method excludes such terms from a constructed feature set which are not associated with at least one example from a set *E*^+^∪*E*^−^. In other words, this feature selection method removes such terms that are highly specific or do not cover any example. This method guarantees that removed terms cannot positively affect the final evaluation score of a rule because these terms cover an empty set of examples. For this reason, if such terms appeared in a rule then the rule would cover an empty set of examples.

#### FS_onlySig

The second feature selection method preserves only features whose terms are significant. P-values are calculated using a Likelihood Ratio Statistic (LRS) as is presented in [20]. The LRS for the two-class problem measures differences between two distributions: the positive and negative class probability distribution within the set of covered examples and the distribution over the whole example set. It is computed as follows:

18 $$\begin{array}{@{}rcl@{}} LRS(r) = 2 \times \left(TP \times log_{2} \frac{\frac{TP}{TP+TN}}{\frac{TP+FN}{|E|}} + TN \times log_{2} \frac{\frac{TN}{TP+TN}}{\frac{FP+TN}{|E|}} \right) \end{array} $$

This measure is distributed approximately as *χ*^2^ distribution with 1 degree of freedom for two classes. If the LRS is above the specific significance threshold then the term is considered to be significant.

#### FS_sigAtLeastOne

The third feature selection method combines the two previous feature selection methods. A term that belongs to the feature set has to satisfy two conditions: 1) that term covers at least one example, and 2) the term is significant which is calculated by the LRS or the term is a generalization of some significant term. This method combines requirements from the previous two selection methods, its selectivity will be experimentally verified later.

### Rule construction

Rule construction is the second step which aims to find a rule that optimizes a given quality criterion in the search space of rules.

The description of the algorithm for single rule learning is depicted in Algorithm 2 where input is a set of positive examples *E*^+^, a set of negative examples *E*^−^, a set of ontologies $\mathcal {O}$, a function *buildMapping* that creates a link between the ontology and the set of examples *E* (*E*=*E*^+^∪*E*^−^), and a parameter *k* that represents the maximal length of induced rules. Note that this function is defined manually by a user. The first step in Algorithm 2 is to find all features. This operation is represented by the function *featureConstruction* at line 4 that assigns all terms from the set of ontologies $\mathcal {O}$ to a set of features $\mathbbm {F}$. To remove irrelevant features from the set of features $\mathbbm {F}$, we propose a function *featureSelection* at line 5. Here, three various feature selection methods are provided as we mentioned in the “Feature selection” section, i.e. *FS_atLeastOne*, *FS_onlySig*, and *FS_sigAtLeastOne*.

The main part of this algorithm is presented in lines 8-24. In this while loop, candidate rules are gradually refined until the maximal length of the rule is reached (*l* variable represents the current length of rule) or there is nothing to refine, i.e. the algorithm did not create any new rule in the previous iteration. In the for loop (lines 11-21), new candidate rules are generated using the application of the refinement operator on the corresponding parental rules. The algorithm iterates over each rule that is presented in the set of rules $\mathbbm {R}$. To this rule, we apply a new ontology-based refinement operator which is represented at line 12 by the function *refineRule* that uses the Redundant Generalization and Redundant Non-potential reduction procedures. Similar to the traditional CN2 refinement operator, the ontology-based refinement operator appends a feature to the refined rule. For example, in the case of a conjuction of terms *R*={*t*1,*t*2,*t*3}, a new rule is created as the union of term *t*4 and terms in rule *R*, i.e. *R*_*new*={*t*1,*t*2,*t*3}∪{*t*4}. A new refinement operator requires the following inputs: rule *r* to refine, a set of features $\mathbbm {F}$, an ontology $\mathcal {O}$ for information about relationships, a score of the best rule $\mathbbm {R}_{BEST\_SCORE}$ that has been discovered, a set of positive and negative examples *E*, and a mapping *M*^′^ that represents a connection between ontologies and examples. The operator returns a set of all refined rules that are not Redundant Generalizations nor Redundant Non-potentials and assigns them to *newCandidates* set.

The *refineRule* function that is described in Algorithm 3 starts with an empty set $\mathbbm {S}$ where a content of this set will be returned at the end of the function at line 10. The cycle from lines 3 to 6 appends every feature to the rule that should be refined. Up to this part, the algorithm is similar to the traditional refinement operator. However, all rules that are not *Redundant Generalization* are excluded from the set $\mathbbm {S}$ using the ontology $\mathcal {O}$ that provides relationships among terms. This is done by calling a function *removeRedundGeneralizations* at line 8. The function *removeRedundNonPotentials* removes such rules that satisfy the definition of *Redundant Non-potential* rules. In this case, the function continuously checks the following: 1) $R \succeq _{r} \forall s \in \mathbbm {S} \cup R$. This is true since each element *s* represents a rule that is created as a refinement of rule *R*. 2) For each *s*, if its potential quality *Q*_*p*_(*s*) is less than the quality $Q(\mathbbm {R}_{BEST})$ then remove *s* and all its more specific rules from the set $\mathbbm {S}$. In other words, all rules in $\mathbbm {S}$ whose potential quality can be greater than the rule with the greatest quality $\mathbbm {R}_{BEST}$ are preserved.

All candidate rules that were generated in *refineRule* function are assigned to the set of new rules $\mathbbm {R}_{new}$. In addition, all *newCandidates* are evaluated by the function *evaluateCandidate* and its corresponding quality score is compared to the rule with the highest quality stored in a $\mathbbm {R}_{BEST\_SCORE}$. If such a compared rule has a better quality then this rule is assigned to the $\mathbbm {R}_{BEST}$ variable and the score is stored in the $\mathbbm {R}_{BEST\_SCORE}$ variable. Simultaneously, the rule has to be significant. To compute this significance, we use LRS as we did in feature selection.

At the end of the algorithm, the best rule of the all rules that have been discovered is returned. If the function *filterRules* at line 22 is omitted then the Algorithm 2 is called a *brute-force exhaustive search* that explores the whole search space and leads to a combinatorial explosion. For this reason, an appropriate heuristics should be provided for reducing the search space. In this work, we use Beam search that expands only the most promising rules based on the evaluation function. Other rules are disregarded. 
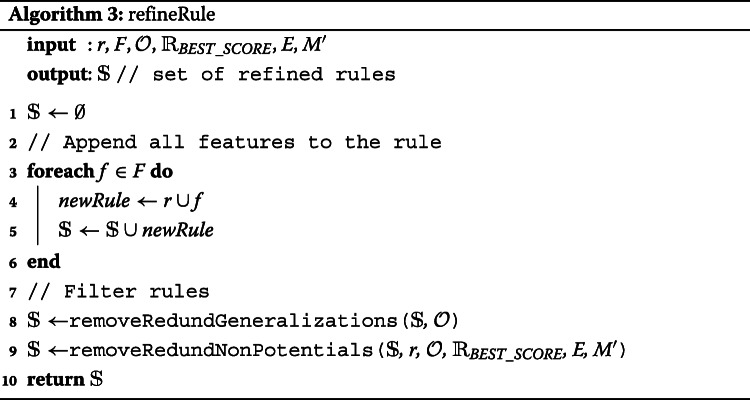


## Results and discussion

In this section, we propose an evaluation procedure that experimentally confirms the efficiency of the new ontology-based refinement operator using two reduction procedures: the *Redundant Generalization* and the *Redundant Non-potential*. The algorithm with the ontology-based operator is called *sem1R* and it is compared against the traditional refinement operator used in CN2, which does not exploit any external knowledge during the rule refining process. Here, it is called *exhaustive refinement*. The ability to reduce a search space is tested on three different datasets with three feature selection methods (*FS_atLeastOne*, *FS_onlySig*, and *FS_sigAtLeastOne*) and with three different evaluation functions (ACC, AUC, and F1-score). Observed parameters as a total number of explored rules, which must be refined to find the best rule, and also run times, were measured for the *sem1R* and *exhaustive refinement*. All presented algorithms are implemented in C++ and work with the Open Biological and Biomedical Ontology (OBO) format. Note that the algorithms require at least one ontology.

Because the proposed algorithm requires three inputs, we define their format as it is used in our R package. The datasets are represented as a two-dimensional binary matrix *D* with *i* rows, *j* columns, a set of row ontologies *R*, and a set of column ontologies *C*. The mapping *M*^′^ is constructed such that each row and column is associated with a subset of ontology terms. This construction step has to be done manually by a user based on expert knowledge. In practice, it is necessary to have specific identifiers for rows and columns and these identifiers are associated with corresponding ontology terms. In gene expression analysis, such an identifier can be gene ID (e.g. FBgn for Drosophila melanogaster, ENSB for human or mouse musculus) for rows and sample ID (e.g. FBbt for anatomy compartments of Drosophila melanogaster or Experimental Factor Ontology for experiment metadata) for columns.

To transform a dataset from a two-dimensional binary matrix to the set of positive and negative examples, we design the following procedure. First of all, we suppose that each element of the matrix *D* represents one example. Then all matrix elements containing 1s are assigned to the set of positive examples *E*^+^ and elements with 0s are assigned to the set of negative examples *E*^−^. For a non-binary matrix *D*, binarization is necessary.

The first tested dataset comes from [30] and describes the gene expression of imaginal discs of Drosophila melanogaster (DISC) where rows of the dataset correspond to genes and columns correspond to samples. Note that this format is used for all tested datasets. Rows (genes) of DISC dataset are described by Gene ontology [6, 7] and KEGG BRITE database. Columns (samples) are described by Drosophila anatomy ontology (DAO) [31]. The second dataset called Dresden Ovary Table (DOT) [32, 33] describes gene expression and RNA localization in fly ovaries using Gene ontology, KEGG BRITE database, and an ontology provided by the authors is freely available at [33], respectively. Note that DOT and DISC are originally formed as a binary matrix. Last but not least, the third dataset was downloaded via Expression Atlas [34] where it is called *Strand-specific RNA-seq of nine mouse tissues*[35] (m2801) and using Gene ontology and Experimental Factor Ontology (EFO) [36]. For binarization, we set up cutoff to 0.5 TPM (Transcripts Per Kilobase Million) because it is presented as a default value in Expression Atlas and it maintains comparable numbers of positive and negative examples. If a value in the matrix is higher than 0.5 TPM then the value is set to 1 and the element is assigned as a positive example otherwise the value is 0 and the element goes to the set of negative examples *E*^−^.

Also, it may be desirable to find descriptive rules only for pre-defined rows (genes) or columns (samples) that are relevant to specific research. Specifically, it can be significantly expressed genes in a treatment group against the control group. In this case, the matrix *D* has only *i*_*s*_ rows corresponding to significantly expressed genes and *j*_*t*_ columns corresponding to samples belonging to the treatment group and *j*_*c*_ columns belong the control group. Here, each of the elements belonging to the treatment group is set up to 1 and is considered to be positive, others are 0 which means negatives. The total number of examples is *i*_*s*_×*j*_*t*_ and *i*_*s*_×*j*_*c*_ for positive and negative examples, respectively.

**Fig. 3:**
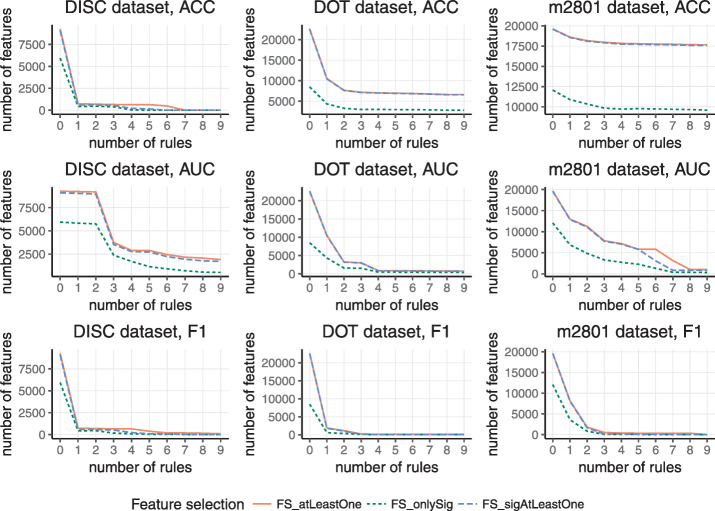
An average number of features across DISC, DOT, and m2801 datasets for three various feature selection methods *FS_atLeastOne*, *FS_onlySig*, and *FS_sigAtLeastOne*. These results were computed using three evaluation functions ACC, AUC, and F1-score

Basic statistics of tested datasets, as a number of rows and columns, a number of positive and negative examples, and a number of ontology terms for given ontologies, are depicted in Table 1. Because there are some terms that do not associate with any example and such terms are not good candidates to be a feature since they do not cover any example, the final feature sets can be given by one of the three feature selection methods mentioned in the “Feature selection” section. The numbers of features that were used for each rule induction step are shown in Fig 3.

**Table 1:** Statistics for DOT, DISC, and m2801 dataset

**Dataset**	**Size**	**# of pos/neg examples**	**# of ontology terms**
DOT	6,510 ×100	309,593/341,407	42,964 (GO)/32,488 (BRITE)/140 (DOT)
DISC	1,207 ×72	65,537/21,367	42,964 (GO)/32,488 (BRITE)/9,255 (DAO)
m2801	12,225 ×26	124,032/193,818	42,964 (GO)/18,786 (EFO)

These experiments clearly confirm our presumptions, defined in the “Feature selection” section, where we assumed that the most reducing feature selection method is *FS_onlySig*. On the other hand, the most benevolent or conservative method is *FS_atLeastOne*, which guarantees that any of the relevant features possibly positively affecting the quality score of the hypothesis will not be discarded from the feature set. The *FS_sigAtLeastOne* demonstrates a similar behavior to *FS_atLeastOne*. Concretely, the *FS_sigAtLeastOne* method produces a smaller feature set than *FS_atLeastOne*. However, the differences are not huge.

To avoid a combinatorial explosion problem in exploring the rule space, we use a Beam search which is represented by *filterRules* function in Algorithm 2. The width of the beam was set no higher than the 100 best rules, the rules are sorted according to their quality score calculated with one of the given evaluation functions. We decided to use this threshold, because greater beam widths result in huge run times in *exhaustive refinement*. Higher beam widths also increase memory requirements. At the same time, the ability of *sem1R* to reduce the search space and consequently reduce run time is obvious even below the beam width of 100. Theoretically, it is anticipated that the ability to reduce a search space grows with the beam width since there are potentially more rules to prune especially for *Redundant Non-potential* procedure.

Total run time and total number of explored rules were observed for rules with the maximum length of 10 because longer rules can be more difficult to interpret in real problems, especially in a biology domain. The total number of induced rules for each dataset was set to 10, for the same reason as previously mentioned. The final results of experiments as total run time in seconds and total number of explored rules are depicted in Table 2 for *sem1R* and in Table 3 for *exhaustive refinement*.

**Table 2:** Total runtime [s] and a total number of explored rules of sem1R algorithm for DOT, DISC, and m2801 dataset

**Dataset**	**Feature selection**	**ACC score**		**F1 score**		**AUC score**	
		**Total time**	**# of rules**	**Total time**	**# of rules**	**Total time**	**# ofrules**
DOT	*FS_atLeastOne*	303.636	107,964	22.381	26,460	142.302	52,638
	*FS_onlySig*	235.947	54,167	11.427	9,780	102.760	25,817
	*FS_sigAtLeastOne*	250.633	107,535	19.813	25,756	115.994	52,136
DISC	*FS_atLeastOne*	10.737	102,219	8.059	178,346	60.780	609,937
	*FS_onlySig*	1.777	87,671	1.109	7,223	33.304	67,558
	*FS_sigAtLeastOne*	1.955	13,041	1.330	11,270	25.861	91,003
m2801	*FS_atLeastOne*	699.273	461,745	28.087	80,079	168.210	225,081
	*FS_onlySig*	914.283	340,039	21.594	18,787	148.992	82,456
	*FS_sigAtLeastOne*	802.176	433,393	18.939	32,054	123.561	124,002

**Table 3:** Total runtime [s] and the total number of explored rules of exhaustive refinement for DOT, DISC, and m2801 dataset

**Dataset**	**Feature selection**	**ACC score**		**F1 score**		**AUC score**	
		**total time**	**# of rules**	**total time**	**# of rules**	**total time**	**# of rules**
DOT	*FS_atLeastOne*	33,800.529	62,192,307	12,807.090	21,977,679	22,814.993	37,604,456
	*FS_onlySig*	15,849.761	30,991,466	5,049.444	8,075,976	9,042.203	15,672,577
	*FS_sigAtLeastOne*	33,638.549	61,912,986	12,743.265	21,674,132	22,726.459	37,176,099
DISC	*FS_atLeastOne*	996.587	10,681,537	881.007	11,017,701	2,214.819	38,874,717
	*FS_onlySig*	623.078	6,125,970	524.412	6,041,883	1,323.920	21,618,242
	*FS_sigAtLeastOne*	963.291	9,406,704	817.055	9,484,372	2,145.880	37,172,305
m2801	*FS_atLeastOne*	53,163.030	153,778,914	6,573.700	26,766,658	12,766.542	64,329,543
	*FS_onlySig*	29,641.080	86,150,004	3,873.421	14,168,195	6,741.368	29,298,233
	*FS_sigAtLeastOne*	53,019.570	153,255,327	6,431.049	25,255,830	12,391.710	59,322,805

**Fig. 4:**
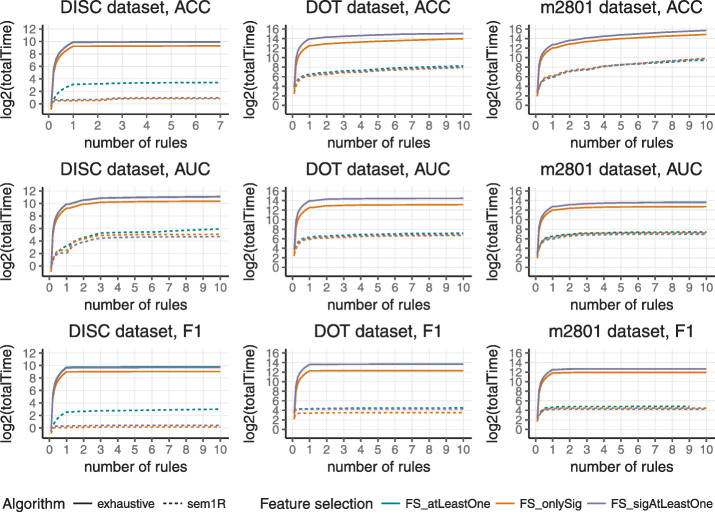
Total run time in logarithmic scale scale depending on the number of induced rules for three datasets (DISC, DOT, and m2801). ACC, AUC, and F1-score were used for evaluating the quality of rules and three feature selection methods (*FS_atLeastOne*, *FS_onlySig*, and *FS_sigAtLeastOne*) were applied before rule induction. Dashed line represents *sem1R*, full line represents *exhaustive refinement*

**Fig. 5:**
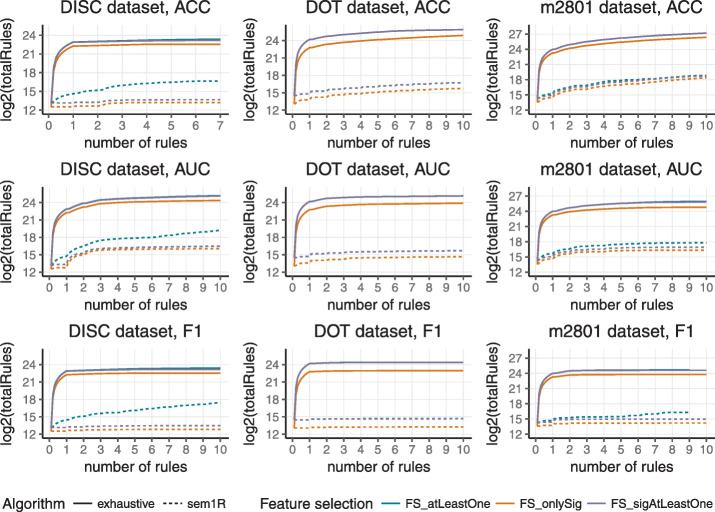
Total number of candidate rules in logarithmic scale depending on the number of induced rules for three datasets (DISC, DOT, and m2801). ACC, AUC, and F1-score were used for evaluating the quality of rules and three feature selection methods (*FS_atLeastOne*, *FS_onlySig*, and *FS_sigAtLeastOne*) were applied before rule induction. Dashed line represents *sem1R*, full line represents *exhaustive refinement*

A graphical representation is shown in Figs. 4 and 5. The first one shows run times in logarithmic scale depending on the number of induced rules for *sem1R* (dashed line) and *exhaustive refinement* (full line). Run time was measured for three datasets with three different evaluation functions and with three different feature selection methods. Evidently, in all cases, the run time of *sem1R* is significantly lower. Figure 5 shows the total number of rules that have been evaluated in a logarithmic scale that depends on the number of rules. As in the previous figure, the number of rules was measured for three datasets with three different evaluation functions and with three different feature selection methods. But even in this case, *sem1R* with its *Redundant Generalization* and *Redundant Non-potential* procedures prunes the rule space more rapidly in comparison with the traditional *exhaustive refinement*. Note that using *FS_onlySig* method, the smallest number of rules is evaluated. This corresponds to the results in Fig. 3.

In all various experimental settings, both *exhaustive refinement* and *sem1R* induce rules with the same quality score across corresponding experiments. The level of significance was set to 99% for feature selection method *FS_onlySig* and *FS_sigAtLeastOne* and also the same significance level for finding the best rule in *induceSingleRule* function. From Figs. 4 and 5 it is obvious that F1-score prunes the search space most and the run of the algorithm is fastest. One of the reasons is that only TP, FP, and FN must be calculated here. On the other hand, AUC is less strict in the pruning of the search space and it is also the slowest, because Eqs. 15, 16 and 17 have to be calculated for every candidate solution and the algorithm has to evaluate the highest number of candidate rules. There is a clear trade-off between the efficiency and complexity of evaluation that stands behind AUC. All results of the experiments are appended to Additional file 1.

For illustration and better understanding, we present an example of 2-terms long rule induced from the DISC dataset, where each term comes from a different ontology. The rule is following: GO:0002181 AND FBbt:00000015. This reported rule is enriched (it covers far more positive examples than expected by random). The FBbt identifier refers to a term from Drosophila anatomy ontology and the GO identifier refers to a term from Gene ontology. In this particular case, the rule says that all genes that are associated with a cytoplasmic translation process (the chemical reactions and pathways resulting in the formation of a protein in the cytoplasm) tend to be over-represented in thorax segment of Drosophila melanogaster.

## Conclusion

We proposed and implemented a new rule learning algorithm that induces a set of rules related to ontologies or taxonomies. Using two novel reduction procedures *Redundant Generalization* and *Redundant Non-potential*, which are part of the proposed ontology-based refinement operator, we dramatically reduce the search space. Consequently, runtime of the algorithm is decreased rapidly as well. These procedures guarantee that any removed rule cannot positively affect the quality of the final hypothesis. Also, three various feature selection methods that help to increase the efficiency of the algorithm were proposed. The algorithm is implemented in C++ and it is available at http://github.com/fmalinka/sem1r as R package. We demonstrated our algorithm on three real gene expression datasets, however, it is generally applicable to any learning task that combines measurements and ontologies, including metabolomics, etc.

## Supplementary information

**Additional file 1** All experiment measurements. Excel file contains all presented measurements for DISC, DOT, and m2801 dataset.

## Data Availability

The datasets generated during and/or analysed during the current study are available at the GitHub repository, [http://github.com/fmalinka/sem1r]
